# Cerebellar Venous Hemangioma: Two Case Reports and Literature Review

**DOI:** 10.3390/jcm13195813

**Published:** 2024-09-28

**Authors:** Biyan Nathanael Harapan, Viktoria Ruf, Jochen Herms, Robert Forbrig, Christian Schichor, Jun Thorsteinsdottir

**Affiliations:** 1 Department of Neurosurgery, LMU University Hospital, LMU Munich, 81377 Munich, Germany; 2 Faculty of Medicine, Center for Neuropathology and Prion Research, 81377 Munich, Germany; 3 Institute of Neuroradiology, LMU University Hospital, LMU Munich, 81377 Munich, Germany

**Keywords:** venous hemangioma, hemangioma, transverse sinus, vascular disorder, vascular malformation

## Abstract

Venous hemangiomas within the central nervous system (CNS) represent a rare pathological entity described by sporadic case reports so far. Comprehensive insights into their histological and imaging features, pathogenesis, natural course, and therapeutic modalities are lacking. This review article presents two patients with contrast-enhancing cerebellar lesions near the tentorium cerebelli lacking edema or diffusion restriction. Despite meticulous preoperative neuroradiological examination, diagnostic classification remained inconclusive. Confronted with both—progressive size and diagnostic uncertainty—surgical intervention was undertaken, resulting in uneventful and complete resection of the lesions. Histopathological analyses subsequently revealed a venous hemangioma in each case. In the literature, the term “hemangioma” is often misapplied and inaccurately used to describe a broad spectrum of vascular anomalies. Therefore, a precise identification is essential since the particular type of vascular anomaly affects its natural course and the treatment options available. We aim to contribute to the understanding of this diagnostically intricate entity by presenting the two cases and by providing a detailed overview of radiological and histopathological features of venous hemangiomas.

## 1. Introduction

Hemangiomas are benign neoplasms of vascular origin characterized by hyperplasia of capillary and venous structures. In the field of vascular malformations, intracranial arteriovenous malformations (AVMs) and cavernomas (cavernous hemangiomas) represent widely encountered and extensively studied differential diagnoses. However, venous hemangiomas must be distinguished from other vascular lesions according to distinctive features in terms of their anatomical, histopathological, and specific imaging characteristics. Despite the abundance of literature on intracranial AVMs and cavernomas, information regarding venous hemangiomas is limited, highlighting the need for further comprehensive investigation [1,2,3,4].

Intracranial AVMs represent abnormal congenital communications between arteries and veins lacking intervening capillary beds due to a maldevelopment of the capillary network [5]. The risk of hemorrhage for AVMs is approximately 3.0% annually, with unruptured AVMs at 2.2% and ruptured AVMs at 4.5% depending on factors such as prior hemorrhage, deep location, venous drainage, aneurysms, and pregnancy [6]. The treatment strategy is determined by the lesion’s size, localization, vascular anatomy, and the presence of hemorrhage. The therapeutic modalities include conservative therapy (e.g., vigilance monitoring and/or administration of antiepileptic drugs), radiosurgery, endovascular embolization, and surgical resection [7]. The selection among these options is based on the individualized characteristics of the AVM, thereby emphasizing a tailored approach to optimizing patient outcome.

Cavernomas are angiographically occult, low-flow vascular malformations. Histologically, cavernomas are composed of endothelium-lined caverns lacking elastic and muscular layers often surrounded by abundant deposits of hemosiderin indicating recurrent leakage of blood [8]. They can be located throughout the central nervous system (CNS), including supratentorial regions, ventricles, brainstem, cerebellum, and the spinal cord. Their manifestation ranges from incidental findings to the presentation of pronounced neurological symptoms, including seizures, focal neurological deficits, and hemorrhagic strokes (annual hemorrhage risk of 0.3% in non-brainstem cavernomas and 2.8% in brainstem cavernomas [9]). The manifestation and severity of these symptoms is dependent on the localization of the cavernoma and the extent of associated hemorrhage [10].

Distinguishing capillary hemangiomas from cavernomas is crucial, given their histological difference and divergent onset with capillary hemangiomas predominantly emerging during childhood and adolescence [11]. Capillary hemangiomas frequently affect the skin and soft tissues, with rare occurrences in the CNS [12]. Microscopic examination typically shows a vascular tumor consisting of nests/lobules of vascular channels that are separated by a variable degree of fibrous bands and lined by well-differentiated endothelial cells [11,13].

Venous hemangiomas are histopathologically characterized by thick-walled blood vessels with smooth muscle and absence of a defined internal elastic lamina [2]. In contrast to cavernomas and capillary hemangiomas, venous hemangiomas emerge as the seemingly most infrequent subtype, with limited reports documented in the existing literature. Consequently, a thorough elucidation of the pathogenesis, natural course, neuroradiological features, and treatment modalities for this particular entity remains unclear.

In this review article, we present two cases of venous hemangiomas, each located adjacent to the right transverse sinus, which are—to our knowledge—the first documented venous hemangiomas in this specific localization. We aim to provide detailed analyses of neuroradiological and histopathological features, and a summary of potential differential diagnoses of intracranial venous hemangiomas.

## 2. Methods

### 2.1. Case Presentation

A comprehensive review of all patients who underwent surgical intervention in our Department of Neurosurgery, LMU University Hospital, Munich, Germany, between January 2010 and June 2024 was conducted, focusing on identifying cases with a confirmed neuropathological diagnosis of venous hemangioma.

### 2.2. Review of the Literature

We performed a literature search using the PubMed database for English-language articles published up until 30 June 2024. Various key terms related to intracranial venous hemangiomas, such as “venous”, “hemangioma”, “venous hemangioma”, “intracranial”, and “brain”, were applied to titles and abstracts. Both authors (BNH and JT) independently reviewed all relevant publications in full to assess their eligibility for inclusion. Studies were excluded primarily based on relevance, specifically if the article did not focus on intracranial venous hemangiomas.

## 3. Results

### 3.1. Case Presentation

Among the cases assessed (between January 2010 and June 2024), only two patients were found to have a histologically verified venous hemangioma, highlighting the rarity of this condition in our patient population.

- Case 1

A 34-year-old female, presenting a medical history marked by recurrent episodes of dizziness and headache, was referred to our department subsequent to undergoing a brain magnetic resonance imaging (MRI) scan. The MRI imaging revealed the presence of a paramedian infratentorial lesion adjacent to the right transverse sinus and tentorium. The initial MRI performed six years ago, prompted by the patient’s complaints of dizziness and neck/shoulder pain, identified the lesion, which has since demonstrated an increase in size.

*Clinical presentation*

The patient presented intermittent hypo-/paresthesia in bilateral lower extremities and myofascial pain syndrome. Neurological assessment revealed no abnormalities.

*Radiographic findings*

An unenhanced computed tomography (CT) scan of the head showed a slightly hyperdense, homogeneous infratentorial mass with a clear delineation broadly adjacent to the tentorium next to the right transverse sinus with a size of 1.5 (anterior-posterior) × 1.4 (left-right) × 1.7 cm (craniocaudal). The lesion did not show any calcifications or signs of hemorrhagic transformation.

**Figure 1:**
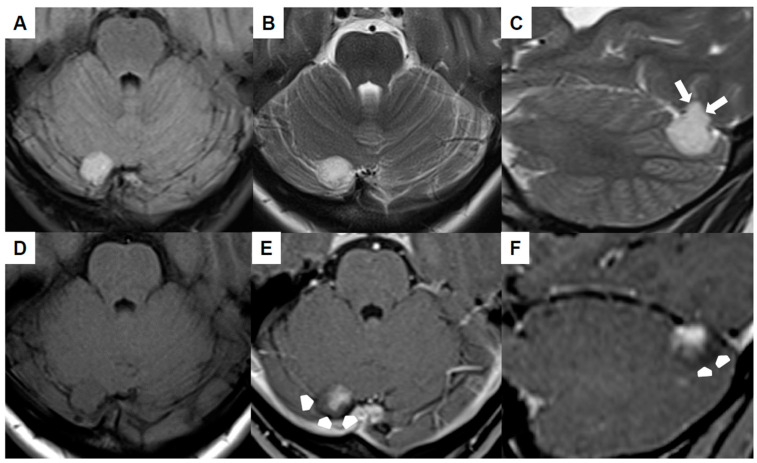
Preoperative magnetic resonance imaging of Case 1: The venous hemangioma showed a hyperintense signal in the fluid-attenuated inversion recovery (**A**) and T2-weighted sequences (**B**,**C**) with signs of tentorial infiltration (arrows in (**C**)). The lesion was cortex-isointense in the unenhanced T1-weighted sequence (**D**) and yielded a relatively homogenous contrast enhancement except for the dorsal margins (arrow-heads in (**E**,**F**)).

Brain MRI (Figure 1A–F) revealed the mass to be homogeneously hyperintense in the fluid-attenuated inversion recovery (FLAIR) as well as T2-weighted sequences and cortex-isointense in the unenhanced T1-weighted sequence. The well-defined lesion displayed no perifocal edema, yet it likely demonstrated infiltration into the tentorium (white arrows in Figure 1C). The differentiation between a primary intra- or extra-axial origin based on MRI was not feasible. Subsequent to the intravenous administration of gadolinium, the lesion exhibited a relatively uniform contrast enhancement, with the exception of the dorsal margins (arrowheads in Figure 1E,F). The adjacent transverse sinus and confluens sinuum did not show any pathological findings, signifying the absence of thrombosis or mass-related pathology. An increase in size was evident in comparison to the initial MRI conducted six years ago.

**Figure 2:**
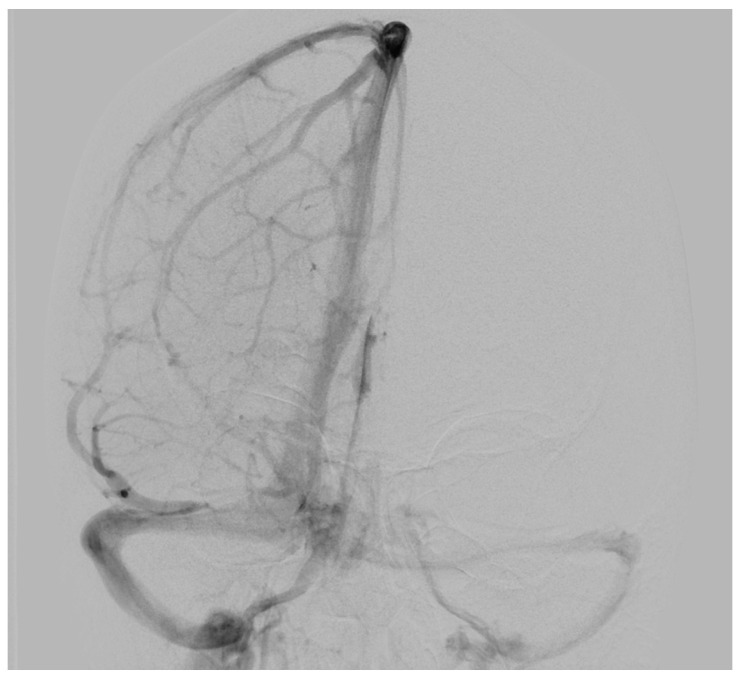
Preoperative digital subtraction angiography of Case 1: The anterior–posterior view showed a dominant venous outflow through the right transverse and sigmoid sinus without signs of infiltration, compression, or thrombosis. No arteriovenous shunt was present.

To determine possible disruption of the venous drainage, a cerebral digital subtraction angiography (DSA) was performed which showed a dominant venous outflow through the right transverse and sigmoid sinus (Figure 2). However, DSA revealed neither any pathological vascularization in the area of the lesion nor further vascular abnormalities such as aneurysms or evident arteriovenous shunts.

*Surgery and postoperative course*

According to the uncertain dignity and increase in size of the lesion, the patient underwent a right paramedian suboccipital craniotomy. The dura was incised caudal to the lesion and retracted toward the transverse sinus. A distinct delineation was observed between the mass and the cerebellum, with the lesion entirely contained within the dura. Macroscopic examination of the mass revealed a well-vascularized, reddish tumor. During surgical preparation, continuous coagulation was applied to reduce the size of the tumor and finally, the lesion could be resected. The postoperative course was uneventful, with a cranial CT on Day 1 post-surgery showing no evidence of space-occupying hemorrhage or demarcation of a territorial infarction. Brain MRI on Day 2 post-surgery confirmed the absence of residual tumor. The patient was discharged without neurological deficits on Day 6.

Three months post-surgery, the patient exhibited no new neurological deficits, and brain MRI indicated no residual mass or recurrence.

*Neuropathological findings*

**Figure 3:**
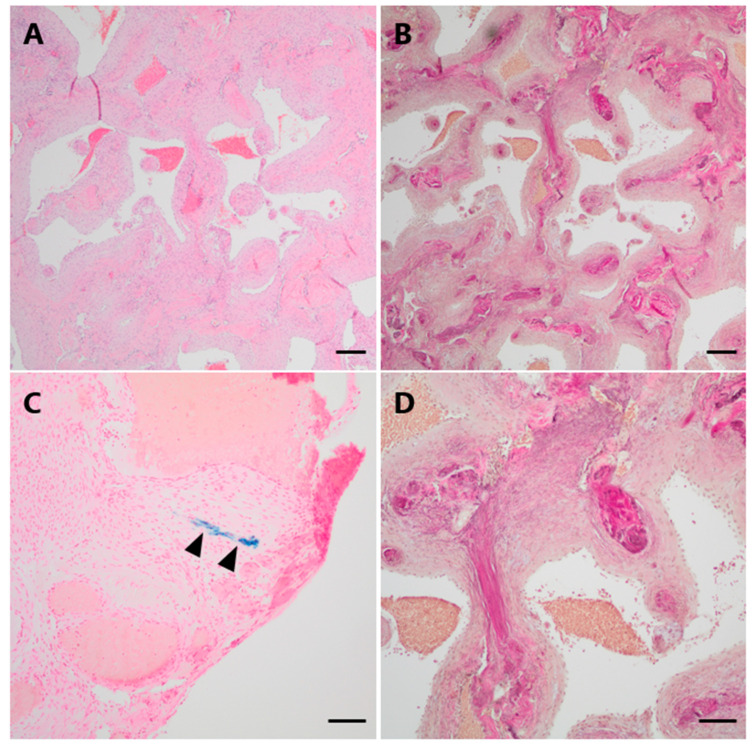
Histopathological examination of Case 1. (**A**) On histological examination, numerous dilated thick-walled blood vessels were seen on H&E. (**B**,**D** (higher magnification)) The venous differentiation characterized by loosely layered elastic fibers without a defined internal elastic membrane is illustrated by an EvG staining. (**C**) Only minimal hemosiderin depositions could be focally observed in a Perls Prussian blue staining (arrowheads). Scale bars: (**A**,**B**): 200 μm; (**C**): 50 μm; (**D**): 100 μm; H&E: hematoxylin and eosin, EvG: van Gieson’s elastica.

Histopathological examination demonstrated a vascular lesion composed of numerous tightly packed thick-walled and variably dilated venous blood vessels with loosely layered elastic fibers but without a distinct internal elastic membrane as a characteristic feature of arterial differentiation (Figure 3A,B,D). Focally, very low amounts of hemosiderin indicating minor blood leakage could be observed (Figure 3C).

The final neuropathological diagnosis was venous hemangioma.

- Case 2

A 52-year-old male presented with an asymptomatic enlargement of the left parotid gland. While an MRI scan revealed unremarkable soft tissues in the neck, the examination incidentally disclosed the presence of an infratentorial mass on the right side. Considering the size and the uncertain dignity, surgical resection was advised.

*Clinical presentation*

The neurological examination was unremarkable, with specific attention to the absence of cerebellar signs such as ataxia, dizziness, and coordination disorders.

*Radiographic and preoperative findings*

**Figure 4:**
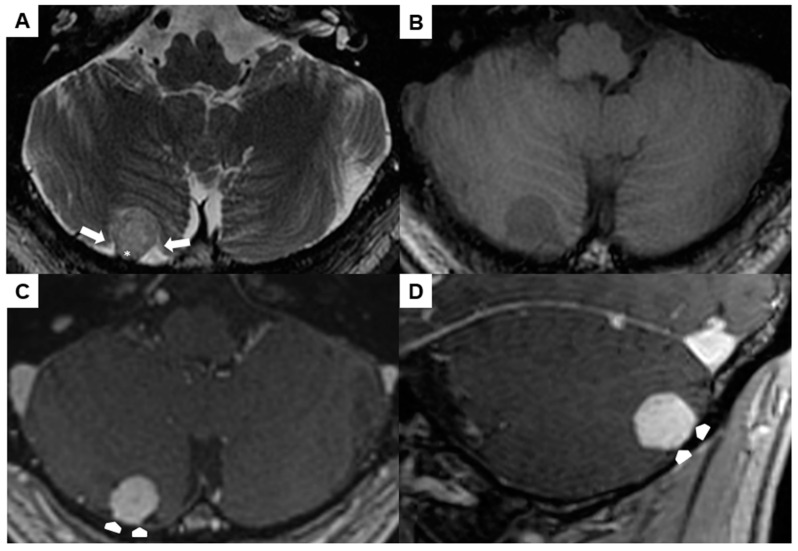
Preoperative magnetic resonance imaging of Case 2: The venous hemangioma showed a slightly hyperintense signal in the T2-weighted sequence (**A**), hypointense signal in the unenhanced T1-weighted sequence (**B**), and distinct contrast enhancement (**C**,**D**). Perifocal cerebrospinal fluid (arrows in (**A**)) and broad dural contact (asterisk in (**A**), arrowheads in (**C**,**D**)) proved the extra-axial location.

A preoperative brain MRI showed a 1.5 × 1.4 × 1.5 cm right-sided infratentorial mass (Figure 4A–D). The spherical lesion exhibited distinct demarcation and was likely situated within the extra-axial compartment, as indicated by the surrounding cerebrospinal fluid and secondary cortical shift (arrows in Figure 4A). It was slightly hyperintense in T2w, hypointense in T1w, and showed a homogeneous contrast-enhancement. No perifocal edema or signs of prior hemorrhage could be observed. The lesion had broad pachymeningeal contact without signs of a “dural tail” (asterisk in Figure 4A and arrowheads in Figure 4C,D).

Due to the dural association, a combined ^68^Ga-DOTATATE positron emission tomography/CT was additionally performed. Hereby, the lesion did not yield increased SSR expression. Hence, diagnosis of a meningioma was very unlikely.

*Surgery and postoperative course*

A right suboccipital craniotomy was performed and ultrasound was used for tumor localization. After dura opening, the tumor was detected with a robust vascularity and attachment to the dura. Small cortical vessels supplying the mass were coagulated. Finally, the tumor including its dural attachment was removed.

Peri- and postoperative course was uneventful. A postoperative cranial CT scan conducted one day post-surgery did not reveal any hematoma or ischemia. The patient was discharged four days post-surgery without any new symptoms. Five months post-surgery, the patient did not present any neurological deficit, and the MRI scan revealed complete resection of the tumor.

*Neuropathological findings*

**Figure 5:**
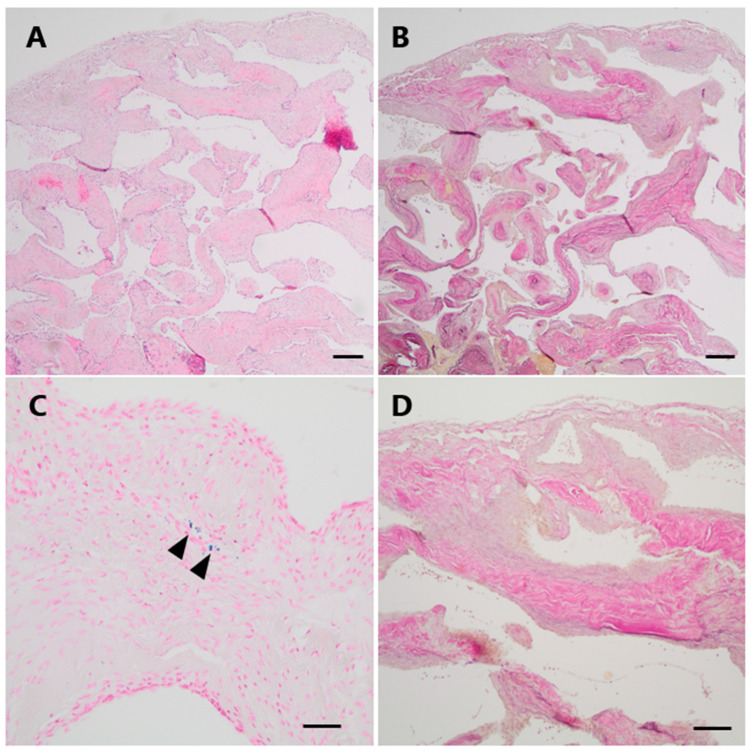
Histopathological examination of Case 2. (**A**) Histological examination revealed a vascular lesion with numerous dilated and thick-walled blood vessels (H&E). (**B**,**D** (higher magnification)) An EvG staning demonstrates the venous differentiation with loosely layered elastic fibers but no distinct elastic internal membrane. (**C**) Only focally, very low amounts of hemosiderin indicating minor leakage of blood could be visualized by a Perls Prussian blue staining (arrowheads). Scale bars: (**A**,**B**): 200 μm; (**C**,**D**): 100 μm; H&E: hematoxylin and eosin, EvG: van Gieson’s elastica.

The histological specimens showed a partially encapsulated convolute of numerous closely packed, vessels of variable size and with thick muscular walls (Figure 5A). Numerous elastic fibers could be demonstrated within the vessel walls on an elastic stain (EvG), but a distinct internal elastic lamina was absent (Figure 5B,D). The Prussian blue reaction yielded only small amounts of focal hemosiderin deposits indicating prior hemorrhage (Figure 5C).

The final neuropathological diagnosis was venous hemangioma.

### 3.2. Review of the Literature

The findings are presented narratively in the discussion and summarized in a table (Table 1).

## 4. Discussion

In both cases, the venous hemangioma adjacent to the transverse sinus was discovered incidentally. Although hemangiomas are generally considered benign, the potential risk for size progression exists, which may result in the compression of adjacent structures, as shown in a case report where a venous hemangioma causes significant compression on the adjacent cerebral peduncle [2].

In previous reports, the localization of venous hemangiomas was variable, e.g., in the internal auditory canal, in the ambient cistern and the optic nerve sheath [1,2,3]. However, occurrences near the transverse sinus have not been reported yet. According to increase in size and uncertainty regarding the dignity, surgical removal was recommended in our presented cases.

Limited information is available regarding the specific (annual) bleeding risk associated with venous hemangiomas. In our presented cases, there were no radiological signs of residual bleeding, and histological examination revealed only minor evidence of hemorrhage. A previous case report described a patient with a ruptured venous hemangioma located within the optic chiasm resulting in subarachnoid hemorrhage and space-occupying hematoma [4]. Neurologically, the patient suffered from progressive loss of vision in the right eye, accompanied by intermittent pulsating headaches, nausea, and vomiting. Postoperative neuroophthalmological assessment demonstrated improved vision, suggesting that surgical intervention may be beneficial for patients with symptomatic and ruptured venous hemangiomas [4]. This case highlights the potential for substantial bleeding risk in association with venous hemangiomas.

The existing literature exhibits partial terminological overlap, complicating diagnostic classification due to microscopic similarities among various vascular malformations and (benign) vascular neoplasms. In alignment with the International Society for the Study of Vascular Anomalies (ISSVA) classification, vascular anomalies are categorized into vascular tumors (neoplastic lesions) and vascular malformations [14]. The morphological resemblance and absence of unique histopathologic features between vascular malformative lesions and vascular neoplasms contribute to diagnostic challenges. True vascular neoplasms are characterized by proliferation and high rates of endothelial cell turnover, while vascular malformative lesions denote localized anomalies resulting from defects in vascular morphogenesis with normal rates of cell turnover. For instance, cerebral cavernous malformations are classified as venous malformations, whereas most hemangiomas are categorized as benign vascular tumors according to the ISSVA classification. By contrast, examples of malignant vascular tumors include angiosarcoma and epithelioid hemangioendothelioma [15]. It is noteworthy that hemangiomas referenced in this classification scheme do not explicitly circumscribe intracranial hemangiomas; rather, they subsume different hemangioma subtypes such as infantile, congenital, spindle-cell, and epithelioid hemangioma.

The ISSVA classification system serves as a valuable framework for differentiating between vascular tumors and vascular malformations. However, it does not specifically include intracranial venous hemangiomas, likely due to their extreme rarity and subsequent lack of systematic analysis. This omission underscores a gap in our understanding and suggests the need for further research. Understanding the biology of vascular malformations is crucial, as it provides insights into their development and behavior, which is essential for accurately classifying these entities [16]. This may have significant implications for understanding their natural progression and determining the most appropriate therapeutic interventions, which can vary greatly depending on the specific type of vascular anomaly [17].

Within this manuscript, a concise compilation of radiological and histopathological characteristics of venous hemangiomas is provided, coupled with an enumeration of their potential differential diagnoses (Table 2 and Table 3). Radiologically, differential diagnoses include cystic meningioma, epidermoid and dermoid tumors, cavernoma, low-grade gliomas, Pacchioni granulations, and other vascular malformations. MRI studies may unveil features that simulate other tumors, intracranial masses, or vascular malformations, rendering the preoperative differentiation of venous hemangiomas challenging. From a histological perspective, considerations must be given to other types of vascular lesions, particularly classical cavernomas or arteriovenous malformations (AVMs), as well as hemangioblastomas or (angiomatous) meningiomas.

Owing to the limited number of cases, available data on treatment options for venous hemangiomas are scarce. A conservative, wait-and-see strategy may be taken into consideration for asymptomatic patients with incidental findings. The case described by Oya et al. [2] and our first case suggest that radiological monitoring is a reasonable approach, particularly when the lesion remains stable in size. However, surgical excision becomes a viable consideration in cases where a progression in size is observed or when a patient is becoming symptomatic by compression of vascular or nerval structures. The efficacy of alternative therapeutic modalities, such as conventional radiotherapy and radiosurgery, remains largely unexplored. Given the lack of crucial information regarding the pathogenesis, natural course, and management of intracranial venous hemangiomas, it is mandatory to collect similar cases in the future to clarify these aspects comprehensively.

In recent years, it has become evident that different types of vascular malformations are linked to inherited and somatic mutations in the PI3K/AKT/mTOR and RAS/RAF/MEK pathways, both of which play a crucial role in cancer biology [18]. This growing understanding has fueled efforts to develop minimally invasive techniques for identifying patients’ mutational profiles, while also investigating how cancer treatments targeting these pathways can be adapted for managing vascular malformations [19]. The emerging field of precision medicine, which focuses on personalized treatment based on genetic insights, is expected to greatly enhance therapeutic options for vascular disorders [20,21].

In our article on venous hemangiomas, a key limitation of the narrative review approach is its broad scope, which can encompass a wide range of studies without the stringent, focused methodology characteristic of systematic reviews. Unlike systematic reviews that use predefined criteria to aggregate and evaluate similar studies, our narrative review offers a general overview. This may reduce the ability to make precise, evidence-based conclusions. However, due to the extreme rarity of venous hemangiomas in the CNS, there are no dedicated systematic studies on this condition; thus, our conclusions are primarily based on our own cases and a selection of other case reports. Consequently, while we aim to offer a comprehensive review, the insights and conclusions drawn are inherently limited by the available evidence and the subjective nature of the narrative review approach.

In summary, we present two cases of venous hemangiomas located adjacent to the right transverse sinus, which, to the best of our knowledge, are the first reported instances of this rare entity occurring in such a specific anatomical location. This unique localization underscores the originality of our findings, as no previously published case reports have documented venous hemangiomas in this region (refer to Table 1 for comparison). Furthermore, our review article offers an in-depth analysis of the radiological and histopathological features of these lesions, providing insights that have not been extensively covered in prior literature. This comprehensive exploration enhances the understanding of the disease entity and its distinct characteristics.

## 5. Conclusions

We present two cases of intracranial venous hemangiomas located near the transverse sinus. Despite thorough neuroradiological examination, a suspected diagnosis could not be defined. Due to the increase in size and diagnostic uncertainty of the lesions, surgical removal was performed resulting in complete resections without any complications. The term “hemangioma” is frequently incorrectly used and applied to a wide range of vascular anomalies. In this review article, we provide a detailed overview of radiological/histopathological features and differential diagnoses of venous hemangiomas. Accurate identification of vascular lesions is crucial, as the specific type of vascular anomaly may influence both its natural progression and the available treatment options.

## Figures and Tables

**Table 1:** Review of the literature of intracranial venous hemangiomas.

Authors	Patient Age	Patient Sex	Location	Symptoms	Signs of Hemorrhage	Observation Period until Surgery	Follow-up after Surgery and Outcome
Our first case	34	F	Extra-axial infratentorial on the right	Dizziness, headache, migraine (unrelated to venous hemangioma/no improvement of symptoms postoperatively)	No	6 years	No recurrence at 3 months, No neurological deficits
Our second case	52	M	Extra-axial infratentorial on the right	Asymptomatic	No	1.5 months	No recurrence at 5 months, No neurological deficits
Oya et al. [2]	32	F	Left ambient cistern	Asymptomatic	No	4 years	Not reported, no neurological deficits
Moore et al. [1]	65	F	Right internal auditory canal	Right-sided hearing loss and vague imbalance	No	-	15 months, transient facial nerve paralysis, headache
Fermaglich et al. [4]	30	M	Suprasellar cistern, intrachiasmal	Progressive vision and visual field loss on the right eye, dyschromatopsia, pulsating headaches, nausea, vomiting	Yes, subarachnoid hemorrhage with blood clots	-	Improvement of central vision
Monin et al. [3]	28	M	Left orbital apex in the optic nerve sheath	Progressive vision and visual field loss on the left eye, dyschromatopsia	No	-	Improvement of visual acuity

**Table 2:** Radiological features of intracranial venous hemangiomas and their differential diagnoses.

Differential Diagnoses	Common Radiological Features
Venous hemangioma	Extra-axial location adjacent to dural structures, distinct contrast-enhancement, absent edema, no diffusion restriction
Cavernoma	Typically intra-axial location, “Popcorn”-like matrix, perifocal hemosiderin rim, distinct blooming (susceptibility-weighted imaging), acute/subacute hemorrhage
Arteriovenous Malformation (AVM)	Intra-axial, nidus, AV-shunt, flow-associated aneurysms, enlarged veins, +/− edema, +/− hemorrhage
Hemangioblastoma	Typically intra-axial tumor of the posterior fossa, nodular contrast-enhancement and cyst, enlarged associated vessels, von Hippel-Lindau syndrome
Meningioma	Extra-axial location with dural tail, singular or multiple lesions, distinct contrast-enhancement, +/− edema, +/− diffusion restriction
Epidermoid cyst	Extra-axial (commonly basal cisterns) and rarely intraosseous location, no contrast-enhancement, absent edema, distinct diffusion restriction
Dermoid cyst	Extra-axial location, no contrast-enhancement, absent edema, no diffusion restriction, fatty signal (hyperintense in T1w and T2w, CT HU -100 - 0), calcifications, risk of rupture and aseptic arachnoiditis/malresorptive hydrocephalus
Low-grade glioma	Intra-axial location, circumscribed or diffuse morphology, no or minor contrast-enhancement, no or minor edema, no diffusion restriction
Pilocytic astrocytoma	Intra-axial location (commonly infratentorial in children and young adults), heterogeneous morphology (mixed enhancing/non-enhancing components and cysts), +/− edema, no diffusion restriction
Arachnoid (Pacchioni) granulation	Association to venous sinus (most frequently within the transverse sinus), T2w-isointense to cerebrospinal fluid, tiny venous structures within the granulation, no contrast-enhancement, no edema, no diffusion restriction

**Table 3:** Histopathological features of intracranial venous hemangiomas and their differential diagnoses.

Differential Diagnoses	Histopathological Features
Venous hemangioma	Closely packed thick-walled vessels of venous differentiation (i.e., without internal elastic membrane), sometimes with venous valves; usually only a sparse amount of hemosiderin is observed
Cavernoma	Densely packed, often dilated blood vessels of variable wall thickness without intervening brain parenchyma (back-to-back) and without distinct venous or arterial differentiation; typically surrounded by substantial amounts of hemosiderin indicating recurrent leakage of blood
Arteriovenous Malformation (AVM)	Tight network of irregular vascular channels of varying wall thickness and size embedded within abnormal brain parenchyma; vessels exhibit at least in part arterial (internal elastic lamina) or venous differentiation; usually no or only minor hemosiderin deposits
Hemangioblastoma	Highly vascularized lesion consisting of stromal cells and abundant thin-walled small blood vessels
Angiomatous (vascular) meningioma	Numerous, partially hyalinized small to medium sized blood vessels that constitute a majority of the tumor mass; intervening tumor cells are sometimes hardly discernible

## Data Availability

Authors can confirm that all relevant data are included in the article. Dataset(s) derived from public resources and made available with the article (references).
